# Intestinal PPARγ signalling is required for sympathetic nervous system activation in response to caloric restriction

**DOI:** 10.1038/srep36937

**Published:** 2016-11-17

**Authors:** Kalina Duszka, Alexandre Picard, Sandrine Ellero-Simatos, Jiapeng Chen, Marianne Defernez, Eeswari Paramalingam, Anna Pigram, Liviu Vanoaica, Cécile Canlet, Paolo Parini, Arjan Narbad, Hervé Guillou, Bernard Thorens, Walter Wahli

**Affiliations:** 1 Lee Kong Chian School of Medicine, Nanyang Technological University, The Academia, 20 College Road, 169856, Singapore; 2 Center for Integrative Genomics, University of Lausanne, Genopode, 1015 Lausanne, Switzerland; 3 Integrative Toxicology and Metabolism, ToxAlim, Research Center in Food Toxicology, National Institute for Agricultural Research (INRA), 180 Chemin de Tournefeuille, 31300 Toulouse, France; 4 Institute of Food Research, Norwich Science Park, Norwich, Norfolk, NR47UA, UK; 5 INRA, UMR 1331 Toxalim, Research Centre in Food Toxicology, Axiom Platform, 180 chemin de Tournefeuille, 31027 Toulouse Cedex, France; 6 Toulouse University, INP, Toxalim, UMR 1331, 31027 Toulouse Cedex, France; 7 MetaToul-MetaboHUB, National Infrastructure of Metabolomics & Fluxomics, 31027 Toulouse Cedex, France; 8 Department of Laboratory Medicine (LABMED), Division of Clinical Chemistry, and Department of Medicine, Metabolism Unit, Karolinska Institutet at Karolinska Universitetssjukhuset Huddinge, SE-14186, Stockholm, Sweden

## Abstract

Nuclear receptor PPARγ has been proven to affect metabolism in multiple tissues, and has received considerable attention for its involvement in colon cancer and inflammatory disease. However, its role in intestinal metabolism has been largely ignored. To investigate this potential aspect of PPARγ function, we submitted intestinal epithelium-specific PPARγ knockout mice (iePPARγKO) to a two-week period of 25% caloric restriction (CR), following which iePPARγKO mice retained more fat than their wild type littermates. In attempting to explain this discrepancy, we analysed the liver, skeletal muscle, intestinal lipid trafficking, and the microbiome, none of which appeared to contribute to the adiposity phenotype. Interestingly, under conditions of CR, iePPARγKO mice failed to activate their sympathetic nervous system (SNS) and increase CR-specific locomotor activity. These KO mice also manifested a defective control of their body temperature, which was overly reduced. Furthermore, the white adipose tissue of iePPARγKO CR mice showed lower levels of both hormone-sensitive lipase, and its phosphorylated form. This would result from impaired SNS signalling and possibly cause reduced lipolysis. We conclude that intestinal epithelium PPARγ plays an essential role in increasing SNS activity under CR conditions, thereby contributing to energy mobilization during metabolically stressful episodes.

Caloric restriction (CR) increases life span and improves the health of diverse species under laboratory conditions^1^,^2^. Lifelong CR can extend lifespans by up to 50% in rodents, with lesser effects should CR commence later in life^3^. CR both delays and slows-down the progression of age-related diseases, including neurodegenerative, autoimmune and cardiovascular disease, type 2 diabetes, and cancer^4^. However, the molecular mechanisms that underlie these beneficial effects remain unknown.

Peroxisome proliferator activated receptor gamma (PPARγ) is a member of the PPAR nuclear receptor subfamily that is primarily known for its insulin sensitizing properties and role as a master regulator of adipogenesis^5^. PPARγ also modulates a wide range of processes including cellular proliferation, differentiation, glucose and lipid metabolism, and inflammation^5^,^6^,^7^,^8^,^9^. PPARγ is highly expressed in adipocytes and in the gastrointestinal (GI) tract, and is expressed at lower levels in the pancreas, liver, and kidney. In the GI tract, PPARγ is present at a relatively high level in the duodenum, and at lower levels throughout the small intestine, reaching its peak expression in the proximal colon, before decreasing again in the distal colon^10^,^11^,^12^. PPARγ expression and activity are induced in the gut by multiple nutrient species, most notably fatty acids and their metabolites, but also by glutamine, curcumin, capsaicin, ginsenosides, vitamin E, and selenium, all of which have anti-inflammatory properties^13^. Importantly, bacterial metabolites and bacterial by-products such as butyrate^14^,^15^, H_2_O_2_^16^, and LPS^17^, also activate PPARγ.

To date, the role of PPARγ in the gastrointestinal tract has, in the main, been restricted to the study of inflammation and cancer, which has led to the identification of PPARγ as a promising therapeutic target in colon cancer. PPARγ inhibits the development of colorectal cancer by decreasing cellular proliferation^18^,^19^,^20^, increasing cell differentiation^18^,^21^, inducing apoptosis^18^,^19^,^22^,^23^,^24^, and by inhibiting angiogenesis^25^. PPARγ agonists also mitigate the symptoms of inflammatory bowel disease, reduce inflammation, and are effective in multiple models of ulcerative colitis^26^,^27^,^28^,^29^,^30^,^31^,^32^,^33^,^34^,^35^, as well as in Crohn’s disease^36^. Moreover, PPARγ is responsible for the selective killing of bacteria associated with IBD^37^. Thus, there is a dynamic balance that involves reciprocal interactions between PPARγ and gut microbiota, with PPARγ both activated by bacteria, and capable of regulating the composition of that intestinal microbiota.

The essential contribution of PPARγ to metabolic regulation in multiple organs suggests potential roles in the intestine other than those already described in inflammation and cancer. We therefore challenged intestinal epithelium-specific PPARγ knockout (iePPARγKO) mice with several metabolic stimuli. Here, for the first time, we provide evidence for a connection between CR and intestinal PPARγ. Under conditions of CR, intestinal epithelial PPARγ increases sympathetic nervous system (SNS) activity and promotes the use of stored fat.

## Results

### Intestinal PPARγ regulates body adiposity in mice subjected to CR

An intestinal epithelium-specific PPARγ knockout mouse was generated by crossing floxed *Pparγ* (PPAR*γ*^fl/fl^)^38^ mice with mice expressing the Cre recombinase transgene under the control of the villin promoter (VillinCre+)^39^. The offspring, PPARγVillinCre+ mice, carry a targeted disruption of *Pparγ* in their intestinal epithelium; these animals, denoted iePPARγKO mice, were used in parallel with littermate controls (PPARγVillinCre-), or wild-type (WT) mice with the same genetic background. Gene deletion was specific to the gastrointestinal tract (see Supplementary Fig. S1), with successful disruption of *Pparγ* resulting in a loss of PPARγ protein expression (Supplementary Fig. S1). A very low level of *Pparγ* deletion was also apparent in the kidney (Supplementary Fig. S1), agreeing with the expression of villin in the epithelial cells of proximal tubules^39^,^40^,^41^. Despite this, we could not detect decreased *Pparγ* mRNA levels in total RNA samples derived from the kidney (Supplementary Fig. S1). No obvious phenotypes were evident in the iePPARγKO mice during *ad libitum* feeding, i.e. there were neither differences in body weight nor the size of internal organs when comparing iePPARγKO and WT mice.

**Figure 1:**
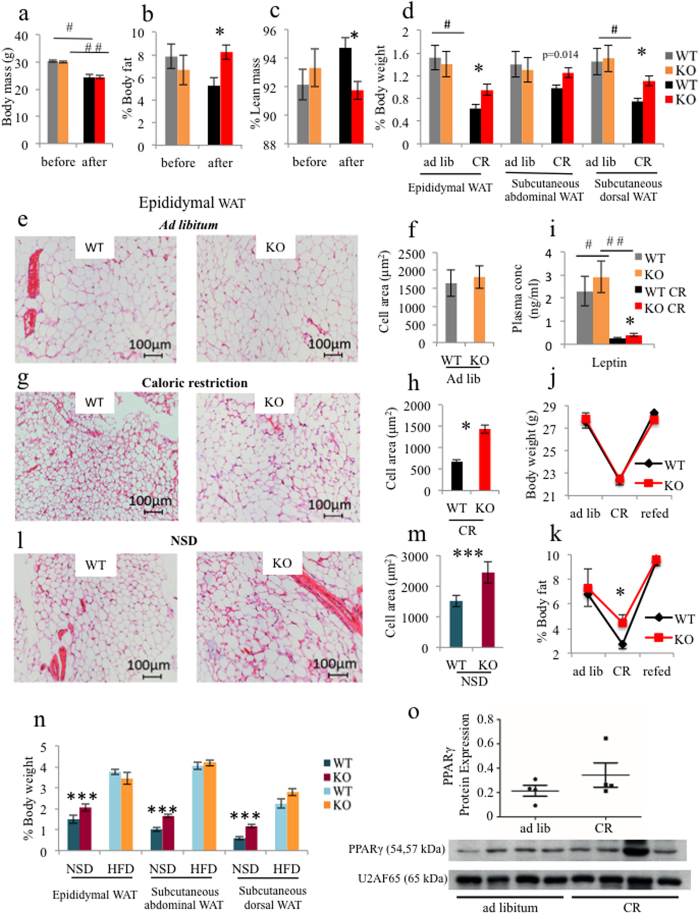
iePPARγKO mice lose less fat than WT mice when under caloric restriction (CR), or fed a no-sucrose diet (NSD). WT and iePPARγKO mice were submitted to CR and their body mass measured before and after the challenge (**a**). Percentage body fat (**b**) and lean mass (**c**) were assayed using EchoMRI, with the pad weights of white adipose tissue (WAT) recorded (n = 10–14 mice) (**d**). Adipocyte size was determined by histological analyses for mice fed *ad libitum* (**e**) and after CR (**g**). The average cell surface area of 50 cells per histologic section (n = 4–5 sections) was determined for mice fed *ad libitum* (**f**) and after CR (**h**). The plasma concentration of leptin was measured for mice fed *ad libitum* and after CR (i); n = 11–14 mice. Body weight (**j**) and the percentage of body fat (**k**) were measured in mice fed *ad libitum*, in mice after 2 weeks of CR, and in mice after 2 weeks of resumption of normal feeding (n = 8–10 mice). Histological sections from the WAT of mice on a no-sucrose diet (NSD) were analysed (**l**), and the average adipocyte surface area measured (**m**). WAT pad weights were measured in mice on a NSD, and a high fat diet (HFD), and are represented as a percentage of body weight (n = 6–16 mice) (**n**). PPARγ protein levels were assayed by western blot (**o**), using tissue derived from intestinal epithelium scrapings of *ad libitum* and CR mice. The signals were quantified, with statistical significance verified using the Student’s t-test; data are presented as means with STD, p = 0.3. All data besides panel o are shown as mean values ± SEM error bar. For panels (f,h,m,j,k,o), the Student’s t test was performed, p < 0.05. For the remainder of the graphs, one-way ANOVA with a Bonferroni post-hoc test was applied. The following symbols ^#,^^##,^*^,^***, correspond to statistically significant differences for the following data sets: WT vs. WT CR; KO vs. KO CR; WT CR vs. KO CR; and WT NSD vs. KO NSD, respectively.

Mice were then exposed to CR, which entailed a 25% reduction in daily food intake for two weeks. As a result of CR, both the iePPARγKO and WT mice lost close to 20% of their body weight (Fig. 1a). Despite having a similar body weight after CR, the remaining body fat content of iePPARγKO CR mice was significantly higher than that of WT CR mice (Fig. 1b). This finding was confirmed by an increased percentage of lean mass identified in WT animals compared to iePPARγKO mice (Fig. 1c), that was not seen in animals fed *ad libitum*. EchoMRI results were confirmed by the weights of white adipose tissue (WAT) pads. Epididymal and subcutaneous dorsal WAT pads were significantly heavier in iePPARγKO CR mice than in WT CR mice (Fig. 1d, expressed as relative (% body weight), and absolute values (gram), Supplementary Fig. S2). Histological examination of epididymal adipocytes showed generally smaller adipocytes in CR versus *ad libitum* mice (Fig. 1e–h). Furthermore, adipocytes from iePPARγKO CR mice were bigger than those from WT CR mice, suggesting reduced lipid release during CR (Fig. 1g,h). However, the adipocytes of iePPARγKO CR mice were smaller than their counterparts fed *ad libitum*, indicating that CR-triggered lipid release still took place in iePPARγKO CR adipocytes, but to a significantly lesser degree than in WT CR adipocytes. This difference in adiposity was reflected by higher plasma leptin levels (Fig. 1i) in CR iePPARγKO mice vs. CR WT mice. We concluded that iePPARγKO mice retain more body fat than WT mice during CR-stimulated energy mobilization.

As a control, we verified that there was no unscheduled deletion of *Pparγ* in epididymal, subcutaneous abdominal, as well as subcutaneous dorsal adipose tissues (Supplementary Fig. S2). Theoretically, gene deletion could occur should the villin promoter be induced under CR stress.

Next we assayed whether the difference in adipose tissue mass between iePPARγKO and WT mice was preserved after resumption of a normal dietary regimen for two additional weeks. During this period, the animals regained weight, to levels comparable to those recorded prior to CR (Fig. 1j), with percent body fat also comparable to pre CR levels in both genotypes (Fig. 1k). Hence, the difference in body fat content in CR conditions was reversible for both iePPARγKO and WT mice.

### Expression of iePPARγKO adiposity phenotype under diverse, prolonged, metabolic stresses

We then tested whether the adiposity phenotype of iePPARγKO mice could be triggered by other situations involving metabolic stress. Mice were given an *ad libitum* no-sucrose diet (NSD; sucrose free diet), or a high-fat diet (HFD) for 6 months. The NSD resulted in an increased mass of subcutaneous and epididymal fat, with enhanced adipocyte size in iePPARγKO mice compared to WT mice (Fig. 1l–n, Supplementary Fig. S2). However, total body mass and food intake were similar in both iePPARγKO and WT mice (Supplementary Fig. S2). Both genotypes increased their fat mass to a comparable degree on the HFD (Fig. 1n, Supplementary Fig. S2). Consequently, we concluded that the iePPARγKO adiposity phenotype only develops in situations of reduced energy availability.

### Metabolic gene expression was unaltered in the WAT of iePPARγKO CR mice

Next we compared changes in gene expression in the epididymal WAT of iePPARγKO CR mice vs. WT CR mice. As CR stimulates lipolysis, and the release of energy stored in adipocytes, we measured the expression of genes associated with lipid metabolism. Acetyl-CoA carboxylase (*Acc*) expression increased under CR compared to *ad libitum* conditions. However, there were no significant differences in the expression of genes related to metabolism, the uncoupling of oxidative phosphorylation, or autophagy, when comparing WAT tissue derived from iePPARγKO CR vs. WT CR mice (Supplementary Fig. S3).

### Liver and muscle are not significantly affected by CR in iePPARγKO mice

We then analysed two other metabolic organs, the liver and skeletal muscle, for any differences between iePPARγKO vs. WT mice under conditions of CR. We found no differences in terms of either liver weight or glycogen content (Supplementary Fig. S3). The expression of major genes associated with lipid metabolism, lipid transport, glycogen synthesis (*Gys2*), and gluconeogenesis were all comparable except for *Pparα* and fructose-bisphosphatase 1 (*Fbp1*), which were upregulated in iePPARγKO CR vs. WT CR mice (Supplementary Fig. S3).

During prolonged fasting, muscles are used as an energy supply, leading to sarcopenia. We analysed muscle mass and found that after the 2-week CR diet, the weight of the soleus, gastrocnemius, and tibialis anterior muscles did not differ between iePPARγKO and WT mice (Supplementary Fig. S3). None of the tested genes involved in muscle fibres, autophagy, and metabolism, showed differential expression in iePPARγKO CR vs. WT CR mice in the soleus and tibialis anterior muscles (Supplementary Fig. S3). Thus, the liver and muscles are not significantly affected in CR iePPARγKO mice. We concluded that CR selectively affects adipose tissue in iePPARγKO mice.

### The iePPARγKO adiposity phenotype is neither the result of altered intestinal lipid uptake nor lipid transport

**Figure 2:**
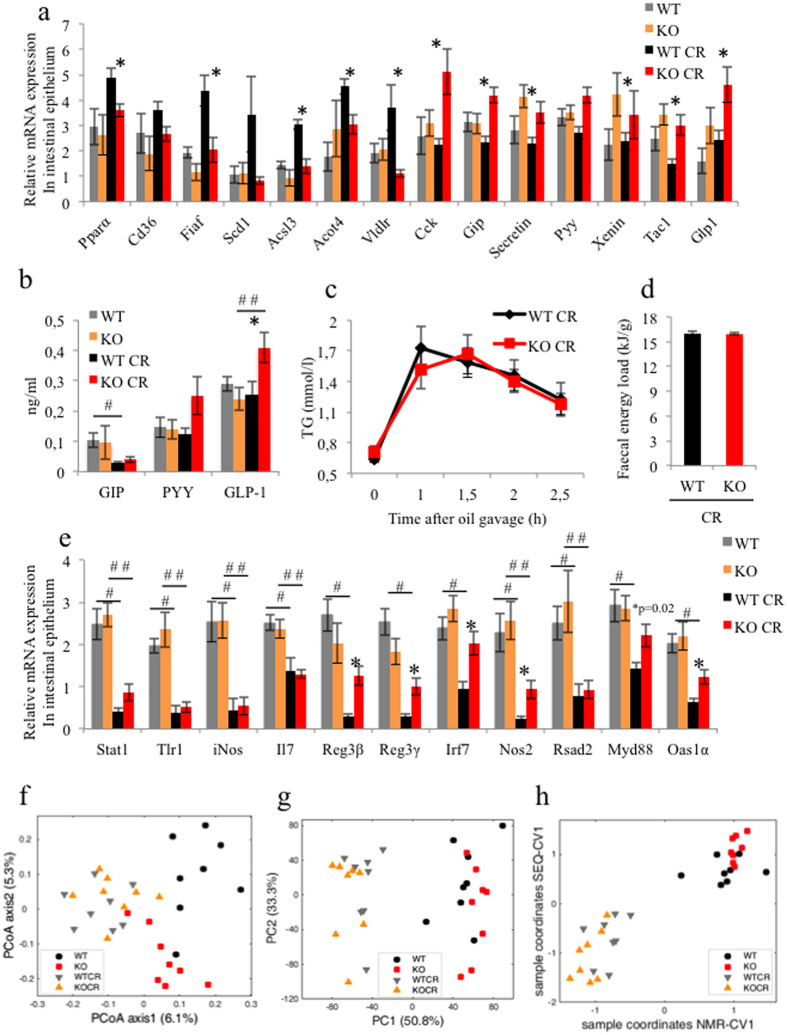
Intestinal lipid uptake and microbiota do not contribute to the iePPARγKO caloric restriction (CR) adipose tissue phenotype. The relative mRNA expression levels of metabolism-associated genes and intestinal hormones were assayed by RT-qPCR in the intestinal epithelium of WT and iePPARγKO mice fed *ad libitum* or under CR (n = 10–12 mice) (**a**). Plasma concentrations of GIP, PYY and GLP-1 were measured for WT and iePPARγKO mice fed *ad libitum* and after CR (**b**); n = 7–9. WT CR mice and iePPARγKO CR mice were gavaged with oil, and their blood triglyceride (TG) concentrations measured at the indicated time points (n = 8 mice) (**c**). The energy content of faeces from WT CR and iePPARγKO CR mice was measured using direct calorimetry (n = 9 mice) (**d**). The relative mRNA expression levels of inflammatory factors and antibacterial and antiviral peptides were quantified in the intestinal epithelium of WT and iePPARγKO CR mice (n = 10–12 mice) (**e**). Murine faecal microbiota composition and metabolites were analysed by sequencing (**f**) and by NMR (**g**), and the two data sets jointly analysed (**h**). The statistical difference in plasma TG and faecal energy load was assessed by the Student’s t-test. Gene expression data were analysed using one-way ANOVA fallowed by Bonferroni post-hoc test. Symbols ^#^, ^# #^, and *, correspond to statistically significant differences between WT and WT CR data, KO and KO CR data, and WT CR and KO CR data, respectively. Error bars depict the standard error.

Since the phenotype observed in iePPARγKO mice is caused by the deletion of *Pparγ* in the intestinal epithelium, we then investigated CR-induced changes in the gut. We observed that PPARγ protein levels were similar between *ad libitum* and CR conditions (Fig. 1o). We then assayed PPARγ transcriptional activity by measuring the expression of several PPARγ target genes (*Pparα, Cd36, Fiaf, Scd1*). While there were no differences between WT and iePPARγKO mice under *ad libitum* feeding conditions, there was a higher expression under CR conditions in WT animals compared to iePPARγKO (Fig. 2a). This result suggests that in the intestine, PPARγ is activated by CR. We also found that the expression of the intestinal hormone genes *Cck*, *Gip*, *Xenin*, *Tac1*, and *Glp1*, were up-regulated in iePPARγKO CR vs. WT CR mice. Consistently, plasma GLP-1 was significantly increased, while there was only a trend for increased PYY and GIP in the iePPARγKO CR compared to WT CR mice (Fig. 2b). In contrast to hormones, the expression of several genes connected with lipid metabolism were upregulated in WT mice in response to CR, but were unaffected in the iePPARγKO epithelium (*Acsl3, Acot4*, and *Vldlr*; Fig. 2a), again demonstrating PPARγ activation under conditions of CR. This suggested a possible differential intestinal lipid uptake in iePPARγKO CR vs. WT CR mice. However, a lipid oral load test revealed no differences in the plasma lipid concentration between iePPARγKO CR and WT CR mice at any time point after oil gavage (Fig. 2c). Furthermore, a direct bomb bomb calorimetry measurement, which assays leftover energy in mouse faeces, showed no differences between the two groups of mice under CR conditions (Fig. 2d). In addition, the quantity of faecal matter generated by iePPARγKO CR and WT CR mice was comparable (Supplementary Fig. S4).

To further investigate whether nutrient uptake was altered in iePPARγKO CR mice, we assayed the levels of various plasma metabolites (see Supplementary Table S1). Triglycerides, cholesterol, and glucose levels differed between the *ad libitum* fed, CR, and NSD groups. However, these levels were comparable in iePPARγKO and WT mice under all experimental conditions (*ad libitum*, CR, and NSD). Similarly, we found no genotype-specific differences in plasma lipoprotein triglyceride content, suggesting that there is no difference between WT and iePPARγKO mice in terms of either hepatic lipoprotein loading or lipid distribution to peripheral organs. Furthermore, levels of plasma lipase activity were unaltered in iePPARγKO mice feeding *ad libitum* vs. CR conditions (Supplementary Fig. S4), suggesting no impact on lipid uptake from plasma by the peripheral organs. We concluded that there is no change in either lipid uptake in the GI tract, or its redistribution to peripheral organs in iePPARγKO mice, and that the iePPARγKO CR adiposity phenotype does not result from the increased uptake of dietary fat.

### The microbiome does not impact the iePPARγKO CR adiposity phenotype

As the expression of inflammatory and antibacterial genes are regulated in the GI tract in response to CR (K. Duszka, in preparation), we next tested whether their expression could also be regulated in the intestines of iePPARγKO mice under CR conditions. We found no difference in the expression of inflammatory factor genes for WT vs. iePPARγKO mice. However, compared to WT mice, the iePPARγKO mouse intestine showed a much weaker down-regulation of antibacterial and antiviral peptide gene expression (*Reg3β, Reg3γ, IRF7, NOS2, Oas1a*) in response to CR (Fig. 2e). Since antibacterial and antiviral peptides influence the composition of the gut microbiota, and intestinal bacteria modify body fat content^42^,^43^,^44^,^45^,^46^, we then performed molecular profiling of the faecal microbiota, which revealed significant differences in the bacterial composition of WT vs. iePPARγKO mice fed *ad libitum* (Fig. 2f, Supplementary Fig. S4, and Supplementary Table S2). Interestingly, CR triggered a strong shift in the faecal microbiota resulting in a similar final microbiota composition in both WT and iePPARγKO mice. These data indicated that the effects of CR on microbiota were stronger than those caused by PPARγ deletion (Fig. 2f). We predicted that these changes would be reflected in faecal metabolites, which we then analysed using nuclear magnetic resonance (NMR). Confirming the sequencing results, there were differences in the faecal metabolite profiles of WT vs. iePPARγKO mice fed *ad libitum* (e.g. lower levels of lactate and succinate in iePPARγKO mice; data not shown). These differences disappeared under conditions of CR, which triggered a large shift in the metabolite profiles in both strains (Fig. 2g).

Integrating the sequencing and NMR datasets using canonical correlation confirmed that CR had a much stronger effect than intestinal PPARγ deletion on gut microbiota (Fig. 2h). In contrast to the significant differences in microbiota composition between WT and iePPARγKO mice fed *ad libitum*, there were no significant differences in either bacterial composition or faecal metabolic profiles in WT vs. iePPARγKO CR mice, suggesting that the gut microbiota was most likely not an important determinant of the iePPARγKO CR adiposity phenotype.

### iePPARγKO mice fail to adjust their body temperature and locomotor activity appropriately during CR

**Figure 3:**
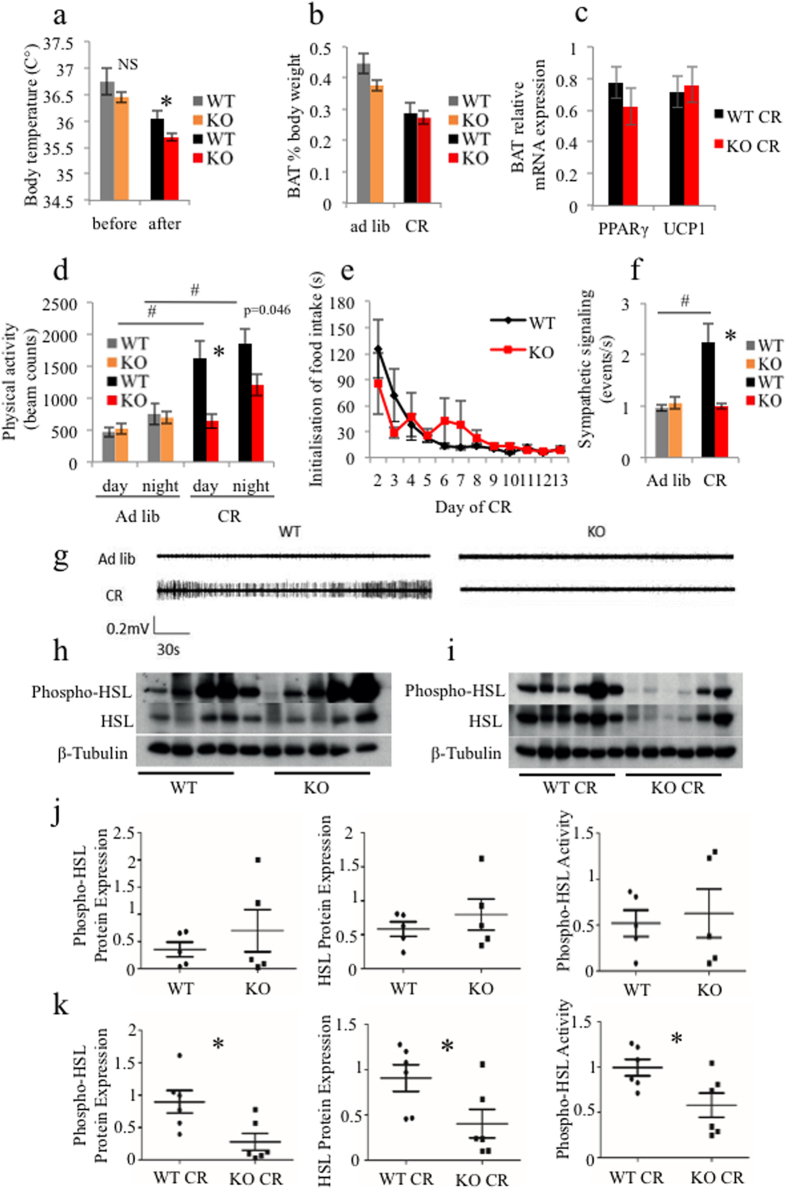
iePPARγKO mice subject to caloric restriction (CR) show altered body temperature, locomotor activity, sympathetic nervous system (SNS) signalling, and WAT lipase expression. Body temperature (n = 7 mice) was recorded before and after CR (**a**). BAT was dissected from *ad libitum* and CR mice with weight (**b**) and gene expression profiles (**c**) subsequently measured. Mice locomotor activity was recorded for *ad libitum* fed mice, and after 14 days of CR (n = 12 mice per group) (**d**). During the daily food portion delivery, the time from when the pellets were placed in the cage to the initiation of feeding was measured each day for the 2-week CR protocol (n = 8–13 mice) (**e**). The frequency of sympathetic nerve firing in WT and iePPARγKO mice fed *ad libitum* or subject to CR was measured (n = 6–7 mice) (**f,g**). Phospho-HSL, HSL, and β-tubulin protein levels in epididymal WAT were assayed by western blot for *ad libitum* (**h**) and CR (**i**) mice. Signals were quantified, with the data submitted to the Student’s t-test and presented as means with STD (**j,k**, respectively). Phospho-HSL activity was determined by normalisation of phospho-HSL protein expression relative to HSL protein expression. *p < 0.05. One-way ANOVA followed by the Bonferroni post-hoc test was used to compare the experimental groups from panels (a–f). Symbols ^#,^^# #,^ * correspond to statistically significant differences between the WT and WT CR datasets, KO and KO CR datasets, and WT CR and KO CR datasets, respectively. Data are presented as means ± SEM.

Next we tested whether the iePPARγKO CR adiposity phenotype was caused by defective regulation of whole body metabolism by measuring metabolic parameters and recording physical activity using indirect calorimetry. Under CR, mice of both genotypes decreased their respiratory exchange ratio values (Supplementary Fig. S4), reflecting the switch from carbohydrate to lipid as a main energy source. WT mice responded to CR by decreasing VCO_2_, although changes to VO_2_ levels were not statistically significant (Supplementary Fig. S4). We detected slight differences in CO_2_ production between iePPARγKO and WT mice only in *ad libitum* conditions, but there were no data to suggest differences in either energy usage or its dissipation between CR WT and CR iePPARγKO mice. Animals of both genotype decreased their heat production upon CR (Supplementary Fig. S4), confirming previous findings for body temperature adjustments to low energy availability^47^. Even though we did not detect differences in heat production between WT and iePPARγKO mice using indirect calorimetry, telemetry revealed decreased body temperatures in iePPARγKO CR compared to WT CR mice (Fig. 3a). Importantly, we neither observed differences in brown adipose tissue (BAT) weight (Fig. 3b), nor *Ucp1* expression (Fig. 3c) when comparing iePPARγKO CR and WT CR mice. Furthermore, *Pparγ* was normally expressed in the BAT of iePPARγKO CR mice, consistent with an absence of *Pparγ* gene deletion (Fig. 3c, Supplementary Fig. S4).

Mice showed increased locomotor activity at night compared to day-time (Fig. 3d). Under conditions of CR, WT mice increased their activity with lesser day/night differences compared to mice feeding *ad libitum*. Movement counts of iePPARγKO CR mice revealed that their mobility was significantly lower than that of WT CR mice and was comparable to that of animals fed *ad libitum*. To evaluate whether this difference in physical activity reflected differences in hunger perception, or a decreased motivation to eat, we measured how fast CR mice reached for food after daily chow portion delivery. As the duration of CR increased, the time taken for all mice to initiate food intake decreased (Fig. 3e), with no difference between iePPARγKO CR and WT CR mice. Furthermore, the expression of hunger-related genes in mouse hypothalami under CR were similar in iePPARγKO and WT mice (Supplementary Fig. S4). Thus, the perception of hunger and the drive to feed do not appear to underlie the differences in physical activity between iePPARγKO CR and WT mice.

We subsequently verified that there was no hypothalamic *Pparγ* deletion in iePPARγKO mice under CR, given that stress could promote ectopic expression of the villin promoter, resulting in a central nervous system-associated phenotype. We confirmed no difference in *Pparγ* expression compared to WT mice (Supplementary Fig. S4), with expression unaffected in the hypothalamus of iePPARγKO mice, and a reproducible absence of *Pparγ* deletion in the hypothalamus (Supplementary Fig. S4).

### iePPARγKO mice show dysfunctional sympathetic nervous system (SNS) stimulation during CR

In seeking a connection between lipolysis stimulation, physical activity, and body temperature, we then recorded autonomic nervous system activity to evaluate the main body function control centre. Interestingly, CR increased SNS firing in WT mice but not in iePPARγKO mice (Fig. 3f,g). SNS sympathetic activity remained at comparable levels in iePPARγKO mice irrespective of CR or *ad libitum* feeding conditions. Furthermore, parasympathetic signalling (PSNS) was upregulated in iePPARγKO CR compared to WT CR mice (Supplementary Fig. S4), which is in line with their less active behaviour.

As the SNS stimulates β-adrenergic signalling in adipocytes, which promotes lipolysis^48^, we then determined levels of hormone-sensitive lipase (HSL) and activated HSL (Phospho-HSL) in epididymal WAT. Although we observed no differences in *Hsl* mRNA expression levels between WT CR and iePPARγKO CR mice (Supplementary Fig. S3), there were decreased levels of HSL and phosphorylated HSL (p-HSL) in the WAT of iePPARγKO CR vs. WT CR (Fig. 3i,k). These differences were not detected in mice fed *ad libitum* (Fig. 3h,j). These results imply that iePPARγKO mice fail to adjust their autonomic nervous system activity in response to CR, which manifests as deficient lipolysis in their WAT.

## Discussion

**Figure 4:**
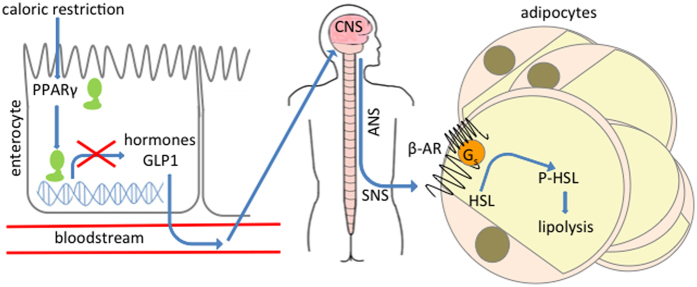
Model of adiposity regulation by intestinal PPARγ during caloric restriction. Transcriptional activity of PPARγ in enterocytes is changed during caloric restriction. This results in decreased expression of incretins and lower plasma level of GLP1. Incretins exported to the bloodstream signal to central nerve system (CNS) and affect the transmission of information to the autonomic nerve system (ANS). In response to shortage of nutrients sympathetic nerve system (SNS) is activated. SNS stimulates β-adrenergic receptors in adipocytes resulting in lipase phosphorylation and lipolysis activation.

Investigating the role of PPARγ in the intestine, we observed that iePPARγKO mice retained more fat than WT mice when subject to a two-week period of CR. Since iePPARγKO and WT mice both lost a substantial portion of their body fat during this period, we propose that the higher WAT mass in iePPARγKO mice after CR results from diminished fat loss, rather than from increased fat accumulation. Supporting this hypothesis, our data showed that iePPARγKO mice manifested no signs of either aberrant lipid uptake or delivery to peripheral tissues, but instead exhibited decreased levels of a key lipolytic enzyme, HSL, and its active form p-HSL, in WAT. From these data we deduced that the iePPARγKO mouse adiposity phenotype results from an altered release of lipids stored in WAT during CR-stimulated energy mobilization. Importantly, the SNS is a major regulator of lipolysis via β-adrenergic receptor activation^49^. Our observations suggest that lipolysis activation is impaired in iePPARγKO mice due to a failure in the SNS response to CR (Fig. 4). Interestingly, the adiposity phenotype is similar for iePPARγKO mice subject to either CR or a NSD, suggesting that some of the benefits of CR might be replicated when the nutritional composition of the diet is changed.

Based on the plasma lipoprotein lipid content, liver weight, and hepatic gene expression data, we conclude that liver lipid metabolism is not affected in iePPARγKO mice and is unlikely to contribute to the adiposity phenotype. Liver glycogen release upon fasting is stimulated by the SNS^50^. However, glycogen stores are activated and depleted in advance of fat deposits, which most likely explains why we saw no differences in these stores when comparing WT CR and iePPARγKO CR mice (i.e. after 2 weeks of CR).

Other than the intestinal epithelium, the villin gene is also selectively expressed in the kidney, in the epithelium of proximal tubules^41^. There are no reports showing villin expression in the juxtaglomerular cells that produce and store renin. Thus, despite our observation of a very limited deletion of *Pparγ* in the kidney, it is extremely unlikely that a renin-angiotensin-aldosterone system failure would contribute to the iePPARγKO adiposity phenotype.

Applying the 2-week CR protocol, we did not observe differences in intestinal epithelium PPARγ protein levels. However, the stimulation of PPARγ target genes indicated an increased transcriptional activity of the receptor during CR, most likely induced by an increase in its endogenous ligands. Under CR, WT mice showed elevated locomotor activity, which is interpreted as natural food-seeking behaviour. Surprisingly, iePPARγKO mice did not show this basic behavioural response to CR. We investigated several possible reasons for this phenotype. First, we tested muscular integrity, since iePPARγKO mice might utilize muscle protein as a source of energy under conditions of CR, which could lead to sarcopenia and diminished locomotive function. However, this was ruled out based on skeletal muscle weight and gene expression data. Second, we tested hunger perception and the motivation to eat. We could find no differences in hypothalamic gene expression, or the time taken to approach food during daily feedings in iePPARγKO CR vs. WT CR mice, demonstrating that neither hunger perception, nor the drive to feed were altered in iePPARγKO mice under CR. Third, we tested activation of the SNS to mobilize energy stores under stress situations. A primitive SNS-related fight-or-flight response is associated with increased mobility to locate food and ensure survival. Critically, the iePPARγKO mice showed a disturbed SNS activation in response to hunger and manifested higher parasympathetic activity compared to WT mice. In addition, their body temperature was significantly lower than that of WT mice. Thus, a decreased perception of energy deprivation might be linked to reduced fat store depletion in iePPARγKO mice. The lack of SNS stimulation in iePPARγKO CR mice and their higher PSNS activity most likely explains why they are less active than WT CR mice.

Lipolysis in adipose tissue is activated by a cascade of phosphorylation events derived from a SNS-stimulated activation of β-adrenergic receptors, leading to lipase activation^48^. Decreased levels of HSL, as well as p-HSL in the WAT of iePPARγKO vs. WT mice under conditions of CR, correlated with reduced SNS activity and the iePPARγKO adiposity phenotype.

Looking for possible traits to link the intestine and autonomic nervous system, we considered extrinsic bacteria-derived, as well as intrinsic, host-specific causes. As bacterial-derived acetate affects the activation of the parasympathetic nervous system^51^, we determined the metabolite composition of mice faeces. However, we could not observe any differences between metabolite generation in WT vs. iePPARγKO mice, including acetates. As a possible host-specific cause of the adiposity phenotype, we then considered intestinal hormones that can influence SNS activity and signalling to the brain^52^,^53^. Again, we could not demonstrate a direct link between intestinal PPARγ and the SNS. However, the observed PPARγ-related modulation of plasma concentrations of intestinal hormones suggests that PPARγ could influence the SNS via intestinal hormone production. This will be the focus of a follow-up investigation.

Previously, Festuccia *et al.*, proposed a link between PPARγ agonist administration and a tissue-specific reduction in BAT sympathetic activity^54^. Here we show, for the first time, that intestinal PPARγ signals the brain, and influences SNS activity. Our results suggest that PPARγ-triggered SNS activation may be WAT specific, as it neither affected BAT, skeletal muscle, nor liver. However, we observed at least two other phenotypes, a decreased body temperature, and reduced physical activity, suggesting the involvement of the central nervous system. Consequently, we anticipate that the iePPARγKO mouse will emerge as a valuable model for research on the gut-brain axis.

Until now, there has been no clear link between PPARγ and CR in any tissue, although a connection has been previously suggested^55^,^56^,^57^,^58^,^59^. We now provide, for the first time, evidence to suggest that intestinal PPARγ is provoked by CR to activate the SNS, triggering a response to energy deprivation. The pathway through which intestinal PPARγ regulates sympathetic signalling remains obscure and is presently under investigation.

## Materials and Methods

### Animal care and experimental procedures

All experiments were performed in accordance with institutional guidelines and were approved by the Vaud Cantonal Authority in Switzerland, and by the Institutional Animal Care and Use Committee in Singapore. PPARγfloxVillinCre+ (iePPARγKO) and PPARγfloxVillinCre- (WT) C57/Bl6/SV129 male mice were kept under a 12 h light/12 h dark cycle. Mice were fed a standard laboratory diet (Diet 3436; Provimi Kliba AG), and housed at a maximum of 5 animals per cage at 23 °C.

The experimental WT and iePPARγKO mice were divided into two groups, the *ad libitum* and CR groups. Mice were housed one per cage to avoid fighting due to food restriction and to exclude the possibility of dominant males taking the majority of the available food. The CR animals were subject to a two-week CR that involved a reduction to 75% of their normal daily food intake. Food was provided daily 1–2 h before the dark phase. All animals had free access to water. Mouse body composition was measured under anaesthesia before and after the CR period using an EchoMRI whole-body composition analyzer (EchoMRI, Huston, TX, USA). Similarly, metabolic parameters (VO_2_, VCO_2_, heat, locomotor activity) were monitored before and after 2 weeks of CR using the Comprehensive Lab Animal Monitoring System (CLAMS, Columbus Instruments, Columbus, OH, USA). Body temperature was assayed using the DSI PhysioTel telemetry system (Data Sciences International, St. Paul, MN, USA). Mice were euthanized using CO_2_, with blood drawn by cardiac puncture. Blood was mixed with 2% aprotinin-EDTA (Sigma Aldrich, St. Louis, MO, USA), centrifuged for 10 min at 8,000 ×g, and stored at −20 °C. White adipose tissue fat pads, liver, and muscle weights were recorded. Tissues were snap frozen and stored at −80 °C until use. The lipid oral load test was performed on CR mice by gavaging 200 μl of oil. Blood was subsequently drawn from the tail at selected time points for analyses.

For the re-feeding experiments, mice were subject to CR for 2 weeks, followed by 2 weeks of free access to food. The weight and body composition of each mouse was measured before, and after 2 weeks of CR, and again after 2 weeks of re-feeding following CR.

For the diet/feeding experiments, 5-week-old mice were switched from standard chow to a sucrose-free diet (no-sucrose diet, NSD; #D12450K), or to a high fat diet from which 60% of energy requirements are derived from fat (#D12492). Both feeds were from Research Diets, Inc. (New Brunswick, NY, USA). Body-fat content was measured by EchoMRI, 16 weeks after the diet regimens began, with mice sacrificed one week later.

### Western blotting

Upon harvest, adipose and duodenal tissues were immediately snap-frozen in liquid nitrogen. Tissues were stored at −80 °C for subsequent lysis and western blot analyses with the indicated antibodies. Adipose tissues were lysed in HNTG buffer comprising 50 mM Hepes, pH 7.5, 150 mM NaCl, 10% glycerol, 1% trition-X-100, supplemented with the cOmplete Mini Protease (Roche #4693159001) and PhosSTOP phosphatase inhibitor cocktails (Roche #4906837001). Tissues were homogenized using a TissueLyser (Qiagen). Lysates were then centrifuged at 18,000 × g, at 4 °C, for 20 min. The fat cake was removed prior to collecting the supernatant. Duodenal tissues were lysed in cold RIPA buffer (Thermofisher Scientific # 89900), again supplemented with the cOmplete™ Mini Protease (Roche #4693159001) and PhosSTOP phosphatase inhibitor cocktails (Roche #4906837001). Tissues were homogenized using a TissueLyser (Qiagen), then subject to centrifugation at 14,000 × g, at 4 °C, for 15 min, before collecting the supernatant. Comparable concentrations of total protein were loaded onto acrylamide/bis-acrylamide gels and, after their electrophoretic resolution, transferred to polyvinylidene fluoride membranes for detection with the indicated antibodies (anti Phospho-HSL (Ser660) antibody, Cell Signaling #4126; anti HSL antibody, Cell Signaling, #4107; anti beta-tubulin antibody, AbCam #ab6046; anti PPARγ antibody (H-100), Santa Cruz #sc-7196; and the anti U2AF65 antibody (H-300), Santa Cruz #sc-48804). Briefly, membranes were incubated with primary antibodies (used at a 1: 1,000 dilution for antibodies purchased from Cell Signaling, or a 1:200 dilution for reagents purchased from Santa Cruz) in 5% BSA/TBST, overnight. Horse radish peroxidase-conjugated secondary antibodies (Goat anti-rabbit IgG-HRP, Santa Cruz #sc-2054) (1: 5,000 dilution) were then added for 1 h. Membrane stripping was with Restore PLUS Western Blot Stripping Buffer (Thermofisher Scientific #46430). Western blots were developed using the Luminata Crescendo Western HRP Substrate (Millipore, #WBLUR0500), with signal detection by X-ray film.

### Plasma and marker analyses

Plasma glucose, lipid, and cholesterol levels were measured using a Hitachi robot (Roche Diagnostics, Basel, Switzerland) according to the manufacturer’s instructions. Plasma lipase activity was analysed using the LPL activity Assay Kit (Roar Biomedical, Inc., New York, NY, USA). Plasma leptin and ghrelin concentrations were estimated using Bioplex (Luminex Corporation, Austin, TX, USA). Liver glycogen content was quantified using a glycogen assay kit (Sigma) according to the manufacturer’s instructions. Faecal energy load was measured using the IKA C200 (IKA Werke, Staufen, Germany) for direct bomb calorimetry. For plasma lipoprotein analyses, the lipoproteins in 2 μL of plasma were separated by size-exclusion chromatography, followed by an online determination of total cholesterol and triglycerides TG as described previously^60^.

### Autonomic nervous system activity

The firing rates of the thoracic branch of the vagal and sympathetic nerves were recorded along the carotid artery as described previously^61^,^62^. The sympathetic and vagal nerves, which lie close to the carotid artery, were dissected free of underlying tissues, to a distance of approximately 5 mm. The nerves were then covered with mineral oil to avoid dehydration and carefully placed on a pair of silver-wire recording electrodes (0.6-mm diameter). The electrodes were connected to a high-impedance probe, and the action potentials displayed and saved by computer after initial amplification through a low-noise amplifier (BIO amplifier, AD Instruments, Oxford, UK). Unipolar nerve activity was recorded continuously for 30 minutes using the LabChart 8 software (AD Instruments). Data were digitized with PowerLab 16/35 (AD Instruments). Signals were amplified 10^5^ times and filtered using a 200/1000-Hz band pass filter. Firing rate analyses were performed using LabChart 8. All animal experimentation protocols were approved by the Vaud Cantonal Authority (authorization VD 2440), Switzerland, and by the Institutional Animal Care and Use Committee (authorization 2015/SHS/1023) in Singapore. The Supplementary Methods provide details for qPCR, the sequencing of 16 S rDNA genes, 1 H NMR metabolomics, and data analyses.

## Additional Information

**How to cite this article**: Duszka, K. *et al.* Intestinal PPARγ signaling is required for sympathetic nervous system activation in response to caloric restriction. *Sci. Rep.* **6**, 36937; doi: 10.1038/srep36937 (2016).

**Publisher’s note**: Springer Nature remains neutral with regard to jurisdictional claims in published maps and institutional affiliations.

## Supplementary Material

Supplementary Information
