# Supplementary material for: Intestinal PPARγ signalling is required for sympathetic nervous system activation in response to caloric restriction
Source: Sci Rep. 2016 Nov 17;6:36937. doi: 10.1038/srep36937 (PMC5113069; doi:10.1038/srep36937)
Supplement: Supplementary Information [file srep36937-s1.pdf]

Intestinal PPARγ signalling is required for sympathetic nervous system activation in response to caloric restriction

Kalina Duszka, Alexandre Picard, Sandrine Ellero-Simatos, Jiapeng Chen, Marianne Defernez, Eeswari Paramalingam, Anna Pigram, Liviu Vanoaica, Cécile Canlet, Paolo Parini, Arjan Narbad, Herve Guillou, Bernard Thorens, Walter Wahli

The Supplementary information file contains:

- Supplementary figures
- Supplementary figures legend
- Supplementary tables
- Supplementary methods

Supplementary Figure 1

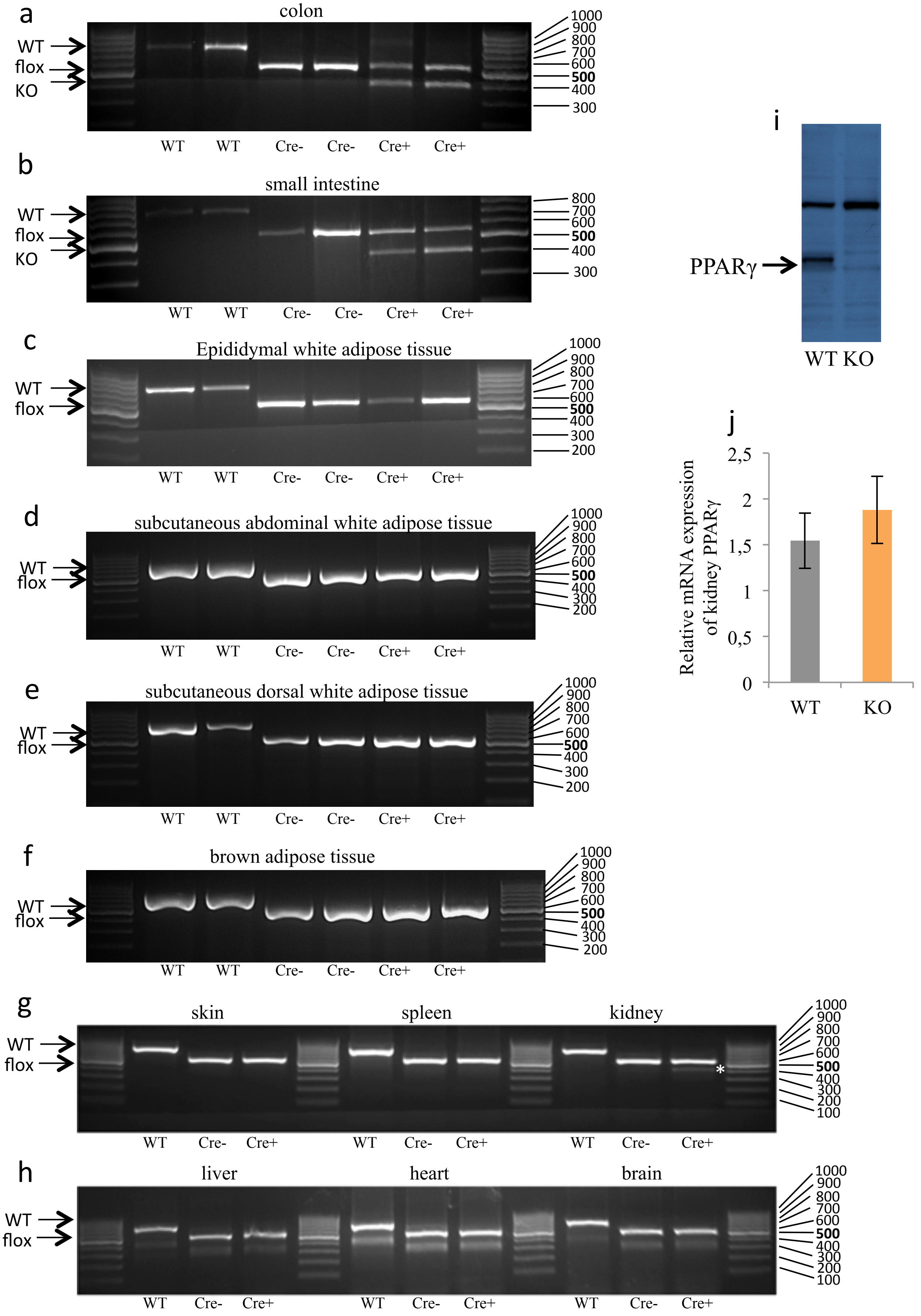

Supplementary Figure 2

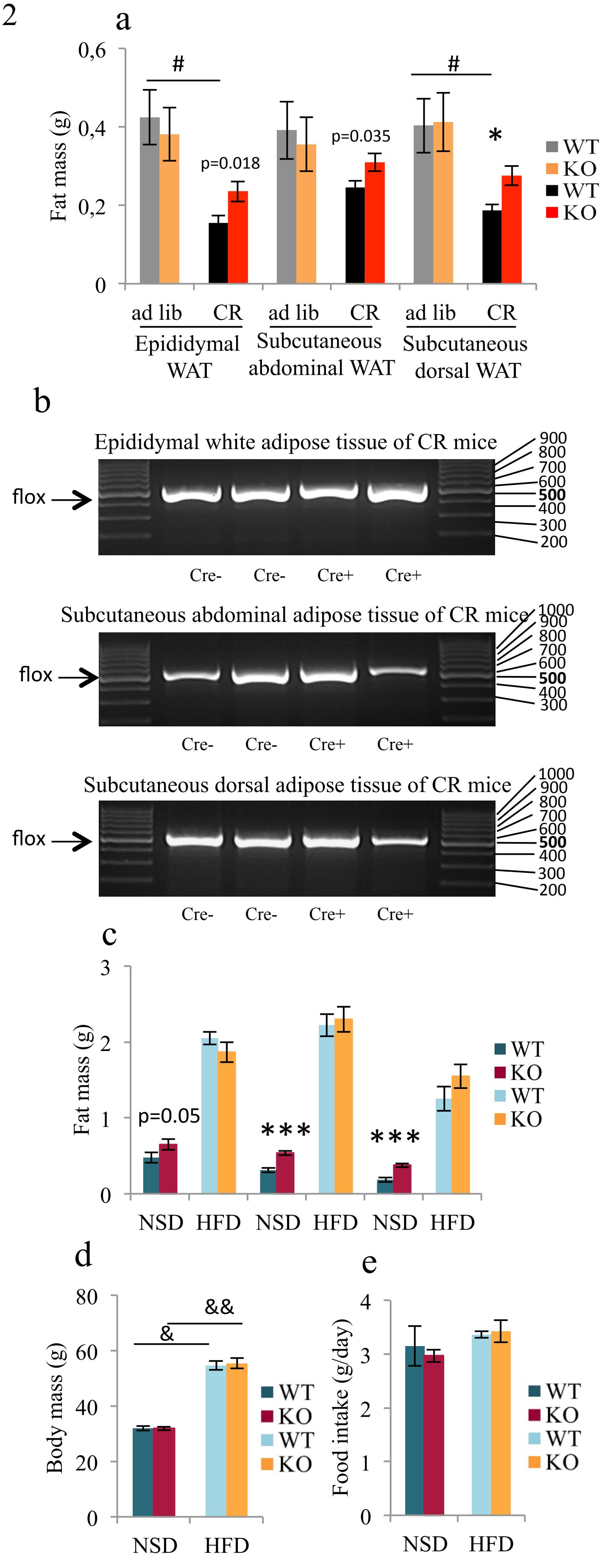

Supplementary Figure 3

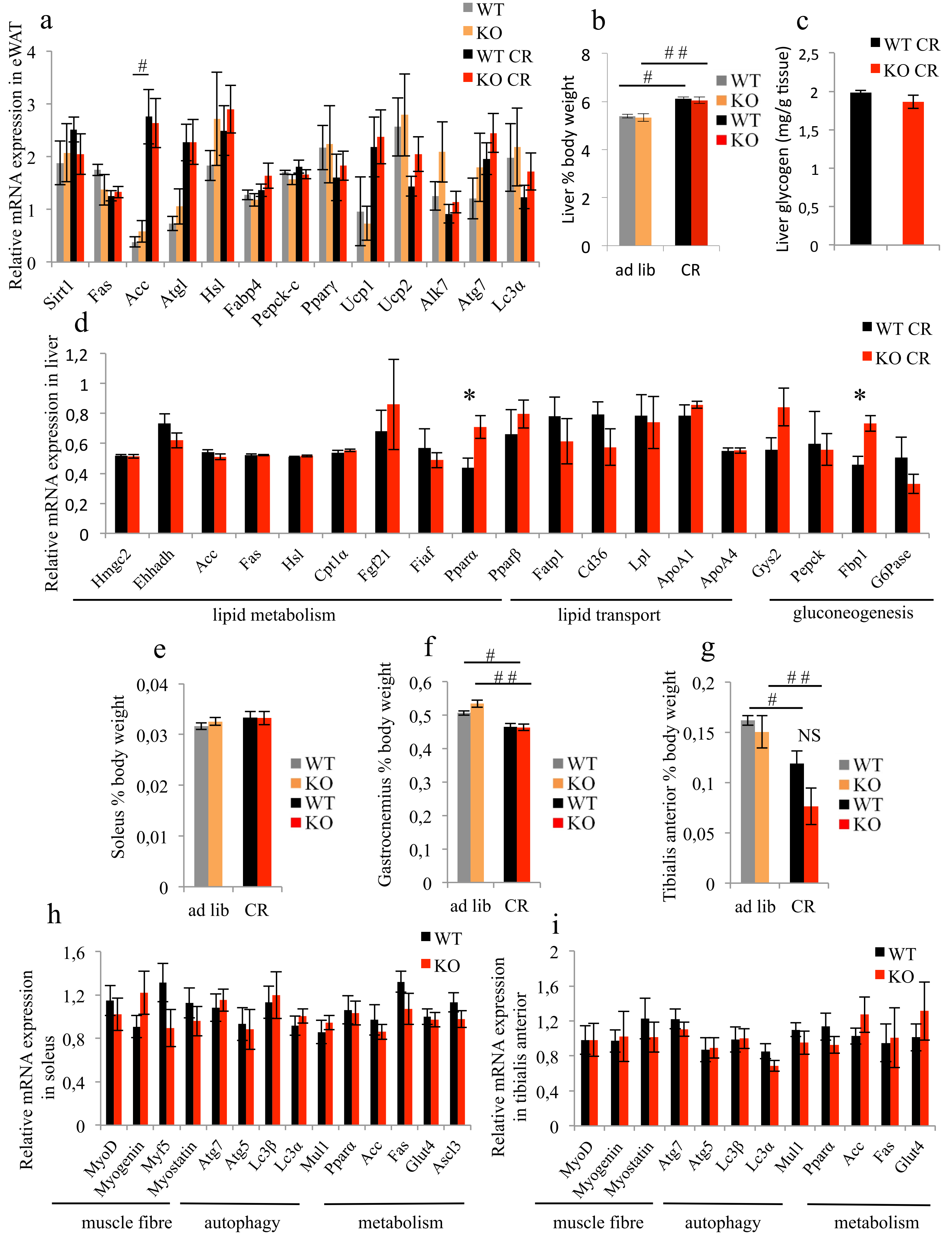

Supplementary Figure 4

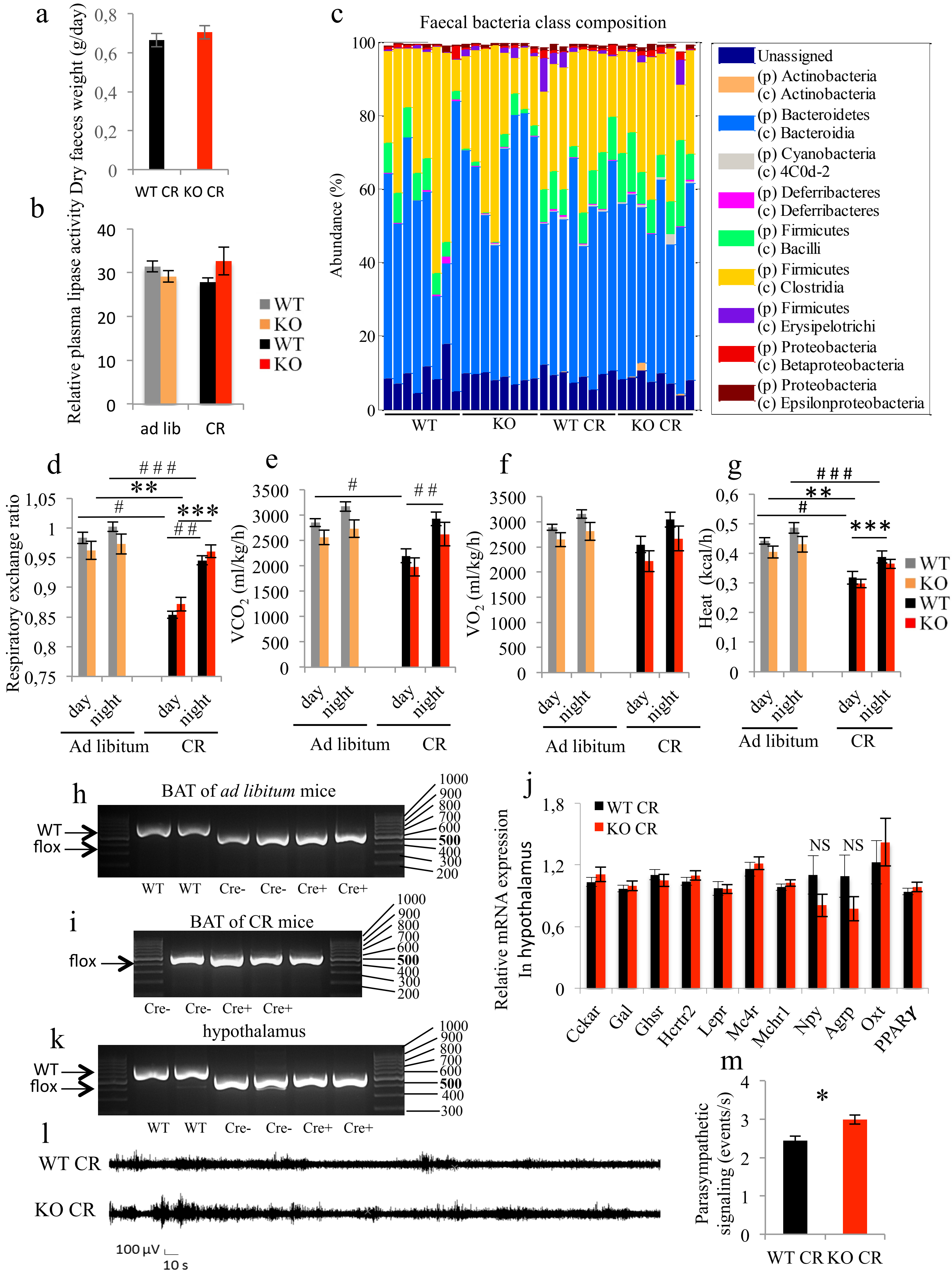

SUPPLEMENTARY FIGURES

Supplementary Figure 1. iePPARγKO mouse genotyping. DNA-based PCR genotyping of the WT, floxed (Cre-), and KO (Cre+) versions of the PPARγ gene, performed for the indicated organs (a-h). Expression levels of the PPARγ protein in the intestinal epithelium of WT and iePPARγKO (KO) mice were measured by Western Blot (i). Kidney mRNA levels of PPARγ were assayed by RT-qPCR using a total organ lysate (j).

Supplementary Figure 2. Body fat content, body mass, food intake, and genotyping data for the WAT of CR mice. White adipose tissue (WAT) pad weights were measured for mice fed *ad libitum*, after CR (a), or after being fed a NSD or HFD (c). DNA-based PCR genotyping of the floxed (Cre-) and KO (Cre+) PPARγ gene were performed using lysates derived from the indicated WAT pads from CR mice (b). NSD and HFD mice fat pad weight (c), body weight (d), and food intake (e) were recorded. One-way ANOVA followed by Bonferroni post-hoc tests were used to compare the experimental groups. The symbols $^{\#}$, *, ***, $^{\&}$, and $^{\&\&}$, correspond to statistically significant differences between the following groups: WT and WT CR, WT CR and KO CR, WT NSD and KO NSD, WT NSD and WT HFD, and KO NSD and KO HFD, respectively. Data are presented as mean ± SEM.

Supplementary Figure 3. Gene expression in the white adipose tissue (WAT), liver tissue, and muscle tissue, as well as muscle and liver weight, in iePPARγKO (KO) and WT mice. Relative mRNA expression levels in epididymal WAT (eWAT) from mice fed *ad libitum* (n = 5 mice), and from mice subject to CR (n = 13 mice), were measured using RT-qPCR (a). Liver weight was recorded and presented as a percentage of total body weight (n = 11–12 mice) (b). Liver glycogen content was also measured (n = 11–12 mice) (c). Relative mRNA gene expression levels in liver were assayed using RT-qPCR (n = 5–7 mice) (d). The weight of the indicated muscle tissue was recorded and shown as a percentage of total body weight (n = 11–12 mice) (e-g). Relative mRNA expression levels in the *soleus* (h), and *tibialis anterior* (i) muscle, were measured (n = 8 mice). Gene expression and organ size data were analysed using one-way ANOVA followed by Bonferroni post-hoc test for panels a, b, and e-g. Two-tail Student's t-tests were performed to verify statistical significance ($p < 0.05$) for the data in panels c, d, h, and i. The symbols $^{\#}$, $^{\#\#}$, and *, correspond to statistically significant differences between the groups: WT and WT CR, KO and KO CR, and WT CR and KO CR, respectively. All data are shown as mean ± SEM.

Supplementary Figure 4. Comparison of the phenotypes of WT and iePPARγKO (KO) mice fed *ad libitum* (ad lib), and after caloric restriction (CR). The weight of dried faeces produced by CR mice over one day was measured (a). Lipase activity was assayed in murine plasma (n = 7–10 mice) (b). Faecal bacterial DNA was sequenced, and

the data presented at class level (c) (n = 7–8 mice; p and c denote phylum and class respectively). Indirect calorimetry measurements were performed during the day and at night for WT and iePPARγKO mice fed *ad libitum* or on a CR diet (n = 12 mice) (d-g). Genotyping was performed using lysates prepared from the BAT of *ad libitum* fed (h) and CR (i) mice using the WT, floxed (Cre-), and KO (Cre+) primers for the PPARγ gene. Relative mRNA expression levels in the hypothalami of CR mice were assayed using RT-qPCR (n = 9-13 mice) (j). DNA-based PCR genotyping was performed on samples harvested from the hypothalamus (k). Parasympathetic nerve firing was recorded for WT CR and iePPARγKO CR mice (n = 7–9) (l, m). One-way ANOVA followed by the Bonferroni post-hoc test was used to compare the experimental groups in panels b and d-g. The two-tail Student's t-test was applied to verify statistical significance ($p < 0.05$) for data in panels a, j, and m; *$p < 0.05$. Symbols $^{\#}$, $^{\#\#}$, $^{\#\#\#}$, ***, and *, correspond to the statistically significant differences found for groups: WT day vs. WT CR day, WT CR day vs. WT CR night, KO day vs. KO CR day, KO CR day vs. KO CR night, and WT CR vs. KO CR.

Supplementary Table 1. Plasma levels of the indicated metabolites were measured in WT and iePPARγKO (KO) mice fed *ad libitum*, under caloric restriction (CR), or fed a no-sucrose diet (NSD).

(mmol/l)	WT	KO	WT CR	KO CR	WT NSD	KO NSD
TG	1.701±0.14	1.827 ±0.23	0.983 ±0.07*	1.156 ±0.10	1.77 ±0.15$^{\#, \Delta}$	1.904 ±0.10$^{\Delta\Delta}$
Free fatty acids	0.639 ±0.05	0.637 ±0.09	0.716 ±0.07	0.72 ±0.10	0.612 ±0.05	0.682 ±0.07
Cholesterol	3.267 ±0.13	2.781 ±0.05	2.171 ±0.18*	2.361 ±0.15**	3.087 ±0.12$^{\Delta}$	3.17 ±0.07$^{\Delta\Delta}$
Glucose	18.09 ±1.00	16.91 ±1.23	11.5 ±0.61*	11.46 ±0.83	18.65 ±0.51$^{\#}$	17.76 ±0.74$^{\#\#}$
VLDL TG	0.998 ±0.14	0.977 ±0.10	0.359 ±0.06*	0.537 ±0.12	0.677 ±0.04	0.648 ±0.06
LDL TG	0.154 ±0.02	0.128 ±0.01	0.088 ±0.01*	0.102 ±0.01	0.118 ±0.01	0.111 ±0.01
HDL TG	0.044 ±0.01	0.043 ±0.01	0.052 ±0.01	0.035 ±0.01	0.033 ±0.01	0.032 ±0.01
VLDL cholesterol	0.202 ±0.03	0.21 ±0.02	0.145 ±0.02	0.16 ±0.02	0.222 ±0.03	0.238 ±0.01
LDL cholesterol	0.303 ±0.04	0.301 ±0.02	0.347 ±0.03	0.321 ±0.02	0.334 ±0.03	0.353 ±0.02
HDL cholesterol	2.282 ±0.11	2.216 ±0.08	2.193 ±0.06	2.17 ±0.11	2.45 ±0.15	2.599 ±0.08$^{\#\#, \Delta\Delta}$

HDL: high-density lipoprotein; LDL: low-density lipoprotein; TG: triglycerides; VLDL: very-low-density lipoprotein. The data were analysed using one-way ANOVA followed by the Bonferroni post-hoc test. The following symbols correspond to statistically significant differences for the indicated datasets: *WT vs. WT CR, **KO vs. KO CR, $^{\#}$WT vs. WT NSD, $^{\#\#}$ KO vs. KO NSD, $^{\Delta}$WT CR vs. WT NDS, $^{\Delta\Delta}$KO CR vs. KO NSD. The metabolite concentrations are represented as the mean ± standard error of the mean.

Supplementary Table 2. Faecal bacterial composition showing the bacterial families that differed significantly in WT and iePPARγKO (KO) mice fed either *ad libitum* or under caloric restriction (CR).

Taxon				Kruskal Wallis p-value (*&** see (a))	Post-hoc tests (with Dunn-Sidak correction) Mean of each group for those found to have statistically significant differences (b)			
Phylum	Order	Class	Family	P	Ad libitum WT vs KO	CR WT vs KO	WT ad lib vs CR	KO ad lib vs CR
Firmicutes	Bacilli	Lactobacillales	Lactobacillaceae	0.0004**				ad lib (1.9) CR (11.1)
Bacteroidetes	Bacteroidia	Bacteroidales	Porphyromonadaceae	0.0005**	WT (2.2) KO (0.3)		ad lib (2.2) CR (0.7)	ad lib (0.3) CR (0.9)
Bacteroidetes	Bacteroidia	Bacteroidales		0.0007**	WT (0.1) KO (0.6)		ad lib (0.1) CR (0.8)	
Cyanobacteria	4C0d-2	YS2		0.0012**			ad lib (0.0) CR (0.4)	
Bacteroidetes	Bacteroidia	Bacteroidales	[Odoribacteraceae]	0.0019**			ad lib (0.1) CR (0.6)	
Firmicutes	Clostridia	Clostridiales	Clostridiaceae	0.0037**		WT (0.2) KO (0.5)	ad lib (0.1) CR (0.6)	ad lib (0.1) CR (0.5)
Firmicutes	Bacilli	Bacillales	Planococcaceae	0.0052**				ad lib (0.0) CR (0.1)
Bacteroidetes	Bacteroidia	Bacteroidales	Prevotellaceae	0.0079*	WT (5.5) KO (14.1)		ad lib (5.5) CR (11.5)	
Firmicutes	Bacilli	Bacillales	Staphylococcaceae	0.0086*				ad lib (0.0) CR (0.2)
Bacteroidetes	Bacteroidia	Bacteroidales	[Paraprevotellaceae]	0.0094*				ad lib (1.2) CR (0.5)
Actinobacteria	Coriobacteriia	Coriobacteriales	Coriobacteriaceae	0.0123*				ad lib (0.1) CR (0.3)
Bacteroidetes	Bacteroidia	Bacteroidales	Rikenellaceae	0.0133*			ad lib (0.7) CR (1.5)	

Statistical test for H_0= "there is no difference between groups for the sequencing abundance data" (a) Kruskal Wallis test for the 4 groups, results shown for families for which Ho was rejected at FDR=10%. P-values were ordered from smallest to largest, and FDR=5% (**) and 10% (*) are indicated. (b) Post-hoc tests on taxons for which Ho was rejected at FDR=10%. The means of each group (%) are indicated for the pairs for which a statistically significant difference is found.

Supplementary methods

qPCR

RNA was isolated from intestinal scrapings and liver tissue using the RNeasy mini kit (Qiagen). Tissue samples were thawed in lysis buffer, immediately teased apart and disrupted using a syringe and needle, and then processed according to the manufacturer's recommendations. RNA was isolated from muscle using Trizol (Life Technologies, Carlsbad, CA, USA). SuperScript II Reverse Transcriptase (Invitrogen, Life Technologies), used with random primers (Promega, Madison, WI, USA), facilitated reverse transcription. Quantitative real-time PCR (qRT-PCR) reactions were conducted using the Applied Biosystems 7900HT system (Life Technologies), with the SYBR green PCR Master Mix (Applied Biosystems, Life Technologies). Statistical tests were performed using the two-sided Student's t-test. Error bars in graphs denote the standard error.

Genotyping and verification of PPARγ deletion

Mouse tissue biopsies were incubated in lysis mix (KAPA Mouse Genotyping Kit, Kapa Biosystems, Inc., Wilmington, MA, USA) according to the manufacturer's instructions. For subsequent PCR, 1 μl of lysate was mixed with KAPA2G Fast Genotyping Mix and the following primers for PPARγ: CCACGTCGTTTAGAATAGCTAGTCCA, GGGACAGACCTCACTAACCCGTAACT, GACCCAGCTCTACAACAGGC; or for VillinCre: GTGTGGGACAGAGAACAAACC, ACATCTTCAGGTTCTGCGGG,

CAAATGTTGCTTGTCTGGTG, and GTCAGTCGAGTGCACAGTTT. The expected PCR product sizes for the PPARγ fragments were 700bp for the wild type allele, 500bp for the floxed allele, and 400bp for the deleted allele.

Sequencing the 16S rDNA genes and meta-taxonomic analyses

Bacterial DNA was extracted from approximately 200 mg of murine faecal material using a FastDNA SPIN Kit for Soil (MP Biomedicals, UK), according to a previously published method [1]. DNA was eluted using 50 μl of DNase- and pyrogen-free DNA elution solution and stored at -20˚C until use. Total DNA was quantified using 1.2 μl of the DNA solution using the NanoDrop ND-1000 UV/vis spectrophotometer (NanoDrop Technologies, Inc., USA) and ND-1000 software (version 3.8.1). PCR amplifications of the V4 and V5 regions of the 16S rDNA gene, and subsequent DNA sequencing, were performed at the Animal Health and Veterinary Laboratories Agency (AHVLA, UK) using the high-throughput GS FLX Titanium platform 454 pyrosequencing platform (the GS FLX Titanium platform), as described by Ellis *et al.*[2].

Bioinformatics analyses of the resulting sequencing reads were performed using the Quantitative Insights Into Microbial Ecology (QIIME) pipeline and the Ribosomal Database Project (RDP) classifier [3,4]. All sequences were filtered to meet the following criteria: read length of 200–1,000bp, a maximum of 6 ambiguous bases, a minimum average quality score of 25 within a 50bp window, and an exact match to the primer sequences. ChimeraSlayer was used to filter trimmed reads for chimeric sequences, with

RDP classifier (version 2.10) used for bacterial taxonomy assignments (with a confidence value threshold of 50%). Trimmed reads (136622 reads in total with means of 5431, 3404, 4644, and 3599 for ad libitum CR, ad libitum KO, CR WT, and CR KO) were clustered into operational taxonomic units (OTUs) at the 97% identity level.

1H NMR metabolomics

Faecal extracts for NMR spectroscopy were prepared by mixing 50 mg of faecal samples with 500 µL of phosphate buffer (0.2 M, pH 7.4) containing 90% D_2O, 1% (w/v) of sodium 3-(trimethylsilyl) propionate (TSP), and 0.3 mM NaN_3. After vortexing, each sample was subject to a freeze−thaw cycle in liquid nitrogen and then homogenized with TissueLyser (QIAGEN, Hilden, Germany) at 20 Hz for 40 s, followed by centrifugation at 1,0000 ×g for 10 min at 4 °C. The supernatants were collected, and the remaining pellet extracted once more as described above. Supernatants obtained from the two extractions were combined and centrifuged at 1,0000 ×g for 10 min at 4 °C. A total of 600 µL of supernatant was transferred to an NMR tube (outer diameter, 5 mm) pending NMR analyses. All ^{1}H NMR spectra were obtained using a Bruker DRX-600-Avance NMR spectrometer (Bruker, Wissembourg, France) with the AXIOM metabolomics platform (MetaToul) operating at 600.13 MHz for ^{1}H resonance frequency using an inverse detection 5-mm ^{1}H-^{13}C-^{15}N cryoprobe attached to a cryoplatform (the preamplifier cooling unit). The ^{1}H NMR spectra were acquired at 300K using the Carr-Purcell-Meiboom-Gill

(CPMG) spin-echo pulse sequence with pre-saturation and a total spin-echo delay (2nτ) of 100 ms. A total of 128 transients were collected into 64,000 data points using a spectral width of 12 ppm, a relaxation delay of 2.5 s, and an acquisition time of 2.28 s. Data were analysed by applying an exponential window function with a 0.3-Hz line broadening prior to Fourier transformation. The resultant spectra were phased, baseline corrected, and calibrated to TSP (δ 0.00) manually using Mnova NMR (v9.0, Mestrelab Research). The spectra were subsequently imported into MatLab (R2014a, MathsWorks, Inc.) All data were analysed using full-resolution spectra. The region containing the water resonance (δ 4.6–5.2ppm) was removed, and the spectra were normalized to the probabilistic quotient[5], and aligned using a previously published function[6].

Analyses of NMR and sequencing data

Taxonomic and NMR data were initially analysed separately using unsupervised multivariate methods in order to assess the compositional similarities between different sample groups, and the relative importance of diet and genotype. For this purpose, the taxonomic data were analysed by PCoA using un-weighted UniFrac distances[7], with NMR data analysed by principal coordinates analysis (PCA) of the mean-centred data. The sequencing abundance data (family level) was also analysed by univariate tests (Kruskal Wallis with FDR=5% and 10%, and post-hoc pairwise tests with Dunn-Sidak multiple test correction)[8,9]. The two datasets were then analysed jointly by canonical correlation analysis (CCA) in order to assess the strength of the relationship between microbiota and

faecal metabolite profiles. To address the high-dimensional nature of the NMR data, it was first reduced by PCA as described above, with the first 5 scores used as input data for CCA. Taxonomic abundance at the family level was used for the sequencing data. Furthermore, the NMR spectral range was limited to the 0.64–4.16-ppm region, with sequencing data limited to 9 bacterial families. This was to avoid issues of sparse data, and to focus on the NMR regions and bacterial families that were most strongly correlated.

References

1 Maukonen, J., Simoes, C. & Saarela, M. The currently used commercial DNA-extraction methods give different results of clostridial and actinobacterial populations derived from human fecal samples. *FEMS Microbiol Ecol* **79**, 697-708, doi:10.1111/j.1574-6941.2011.01257.x (2012).

2 Ellis, R. J. *et al.* Comparison of the distal gut microbiota from people and animals in Africa. *PloS one* **8**, e54783, doi:10.1371/journal.pone.0054783 (2013).

3 Caporaso, J. G. *et al.* QIIME allows analysis of high-throughput community sequencing data. *Nature Methods* **7**, 335-336, doi:10.1038/nmeth.f.303 (2010).

4 Wang, Q., Garrity, G. M., Tiedje, J. M. & Cole, J. R. Naive Bayesian classifier for rapid assignment of rRNA sequences into the new bacterial taxonomy. *Applied and Environmental Microbiology* **73**, 5261-5267, doi:10.1128/aem.00062-07 (2007).

5 Dieterle, F., Ross, A., Schlotterbeck, G. & Senn, H. Probabilistic quotient normalization

as robust method to account for dilution of complex biological mixtures. Application in 1H NMR metabonomics. *Anal Chem* **78**, 4281-4290, doi:10.1021/ac051632c (2006).

6 Veselkov, K. A. *et al.* Recursive segment-wise peak alignment of biological (1)h NMR spectra for improved metabolic biomarker recovery. *Anal Chem* **81**, 56-66, doi:10.1021/ac8011544 (2009).

7 Lozupone, C., Hamady, M. & Knight, R. UniFrac--an online tool for comparing microbial community diversity in a phylogenetic context. *BMC Bioinformatics* **7**, 371, doi:10.1186/1471-2105-7-371 (2006).

8 Dunn, O. J. Multiple Comparisons Using Rank Sums. *Technometrics* **6**, 241-252, doi:10.1080/00401706.1964.10490181 (1964).

9 Benjamini, Y. H., Y. Controlling the False Discovery Rate: A Practical and Powerful Approach to Multiple Testing. *Journal of the Royal Statistical Society* **57**, 289-300, doi:doi:10.2307/2346101 (1995).
